# Musculoskeletal care – at the confluence of data science, sensors, engineering, and computation

**DOI:** 10.1186/s12891-022-05126-x

**Published:** 2022-02-22

**Authors:** Suchitra Kataria, Vinod Ravindran

**Affiliations:** 1 Melange Communications Pte Ltd, Singapore, Singapore; 2 Centre for Rheumatology, Calicut, Kerala 673009 India

**Keywords:** Human data, Data analytics, Artificial intelligence, 3D printing, Prosthetics, Patient-reported outcomes, Exoskeleton, Research, Connected care, Internet of medical things, Population health, Personalised care, Patient care, Clinical trials

## Abstract

Data has always been integral to modern medicine in almost all aspects of patient care and the recent proliferation of data has opened up innumerable opportunities for all the stakeholders in trying to improve the quality of care and health outcomes including quality of life and rehabilitation. Greater usage and adoption of digital technologies have led to the convergence of health data in different forms – clinical, self-reported, electronic health records social media, etc. The application and utilization of patient data set continue to get broadened each day with greater availability and access. These are empowering newer cutting-edge solutions such as connected care and artificial intelligence, 3D printing and real-life mimicking prosthetics. The availability of data at micro and macro levels has the potential to act as a catalyst for personalized care based on behavioral, cultural, genetic, and psychological needs for patients with musculoskeletal disorders. Realistic algorithms coupled with biomarkers which can identify relevant interventions and alert the care providers regarding any deterioration. Although in the nascent stage currently, 3D printing, exoskeletons, and virtual rehabilitation hold tremendous potential of cost-effective, precise interventions for the patients.

## Background

Human Data Science (Health Big Data) – an amalgam of data science and technology with bioscience is providing new insights to our understanding of human health. Human data comprising social and environmental milieu around individuals and their interrelationship with the health system, biological processes in human health, and dynamic analysis of these information pieces result in vital information. The importance of human data science and its applicability has been highlighted by the ongoing Covid-19 pandemic where it resulted in making available various approved vaccine candidates [1] in less than 12 months after the official declaration of the pandemic by the World Health Organization (WHO) [2]. Human data science has immense promise for optimizing health system performance, disease prevention and treatment, improving drug safety, and enhancing prognosis through data modeling response prediction. Global health – data-science initiative is a global data repository with open access to real-time epidemiological anonymized line list data. Its Covid-19 dataset contains 10 million anonymized cases from over 100 countries. The database includes up to 40 associated variables for each patient [3]. Such open and easy-to-use datasets are of immense use for epidemiologists, researchers, and policymakers. This review intends to shed light on the development and impact of data-driven care in the realms of rheumatic musculoskeletal diseases (RMDs) care [4].

### Search strategy

Our literature search covered the Medline, Embase, Scopus, Web of Science, and Google Scholar databases and included articles published in English between January 1980 to June 2021, but we did not intend to ignore any high-quality relevant earlier literature. The following MeSH terms/keywords were used Big data, Data Science, Rheumatic Musculoskeletal Diseases, Digital Divide, Health Service Accessibility, Mobile Applications, Health Records, Artificial Intelligence, Machine Learning, Exoskeleton Devices, Wearable Devices. Our search underscored a dearth of “conventional"  published matter in an area that is evolving fast. Therefore, to bring the readers up to date with this topic, in addition, we have used several contemporary online resources.

## Results

### Human data science – foundation, gaps, and benefits

**Fig. 1:**
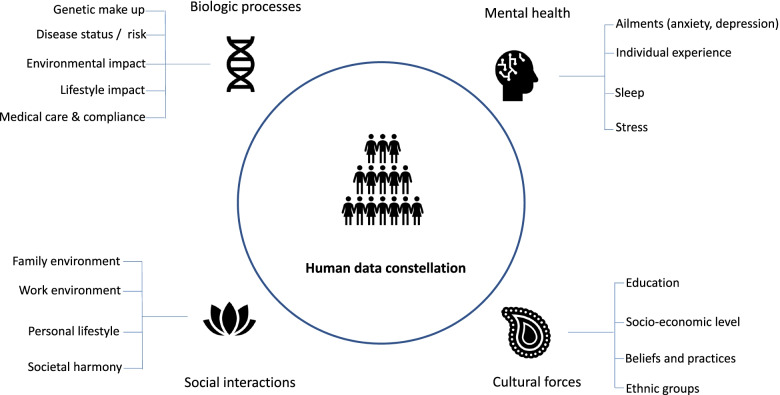
Human data constellation. From authors own source, made using licensed version of MS Powerpoint

The fundamental blocks to the human data science are – availability of disease-specific big patient data sets, application of data science techniques, cyber security and data privacy infrastructure, the proactive and supportive regulatory environment for innovative usage, translational research to unearth disease processes, and technology backbone to maximize the potential of machine learning and artificial intelligence. An increasing amount of data in the healthcare environment derived from multiple sources helps in understanding disease processes at the molecular level, expands the ability to fight diseases, maintain good health and develop new treatments to address unmet needs. Figure 1 illustrates the key macro factors responsible for data diversity, personal genetic signature, and day-to-day impact on variability.

Besides the evolution of newer data types like genomic data, biomarkers, digital data enablers like electronic health records (EHR), insurance claims, wearable sensors, online surveys, variety of other digital tools are generating and collecting healthcare data more than ever before. Data metamorphosis involving collection, standardization, linking datasets & formalization, however, are the biggest challenges. Most often the datasets are not uniform with apparent gaps in data collection, data quality, and data bias. With the further evolution of artificial intelligence and machine learning, there will be a need for more elaborate and diverse datasets adhering to quality standards, appropriateness, and relevance. Our recommendations to overcome these anomalies and harness the untapped potential of digital healthcare benefitting all the stakeholders have been presented in Table 1.

**Table 1:** Measures to improve data collection and data quality and overcome data bias

• Implementation of accessible EHRs with interoperability.	
• Investment and implementation of simple, low-cost digital technologies.	
• Dynamic consent, privacy, and data security processes with IT backbone for data access and sharing.	
• Strong governance and proactive policies for digitization at all levels including workforce facilitating patient engagement.	
• Foster digital literacy and develop leadership.	

**Fig. 2:**
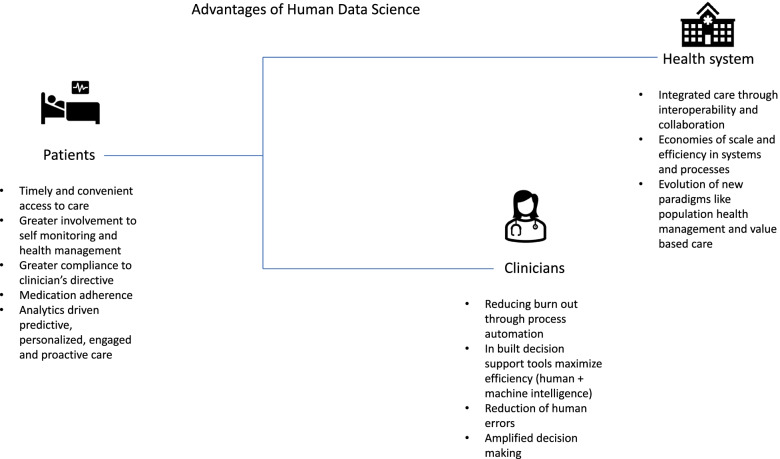
Advantages of human data science. From authors own source, made using licensed version of MS Powerpoint

It is also important to remember that data-driven digital care is not about the adoption of technologies but the change in culture with processes to benefit the patients, clinicians, and healthcare system on the whole. For example, various medical devices, their data flowing onto information technology (IT) systems powered by software-driven by connected technologies in the health system milieu ensuring services go on to create something called the Internet of Medical things (IoMT). This IoMT is the heart of the healthcare renaissance being applied in various forms enabling patient diagnosis, monitoring, treatment, and management with greater efficiency [5]. Figure 2 illustrates the main advantages of human data science to its, key stakeholders.

Marabita et al. collected serial clinical measurements, health surveys, genomics, proteomics, autoantibodies, metabolomics, and gut microbiome data from 96 individuals who participated in a data-driven health coaching program for a 16-month duration with continuous digital monitoring of activity and sleep. The research group generated > 20,000 biological samples and a compendium of > 53 million primary data points for 558,032 distinct features from this study. Multiomics coupled with unsupervised, data-driven digital health measurements provided an integrated view of human health’s distinct and independent molecular factors linked to obesity, diabetes, liver function, cardiovascular disease, inflammation, immunity, exercise, diet, and hormonal effects [6].

The importance of data granularity can be gauged from the Northwestern University-led research team’s approach in developing a novel skin-mounted sticker or epidermal microfluidic device [7]. This sticker absorbs sweat and then changes colour to provide an accurate, easy-to-infer diagnosis of cystic fibrosis in a matter of a few minutes. The sticker is comfortable and imperceptible to the wearer and collects 33% more sweat than current clinical methods ensuring that one test will consistently collect a large enough sample to provide an accurate result. The in-built colorimetric sensors detect measure, analyze and provide a quantitative assessment of chloride concentrations in real-time through a smartphone camera. The researchers believe that sweat stickers could be potentially used to routinely monitor cystic fibrosis patients’ long-term health and track their response to treatment. Provided the bio-sticker achieves clinical validation, cystic fibrosis patients under treatment could potentially use the sticker in the confines of their homes to track their symptoms and hydration levels. Data is the chief driver here which enables quick diagnosis opening up interesting opportunities in patient care, disease outcomes, quality of life (QoL), cost savings, and most important patient convenience.

### Population health management

Population health identifies the needs of the population (public health) through big data, patient engagement and health, and care delivery. The goals are to translate the advantages into pragmatic applications and derive advantages. Clustering the population according to the disease status, severity grade, geography, ethnic groups, socioeconomic strata to identify risk factors can help in deploying and allocating resources for addressing health inequalities. The data stream coming out of various patient sources results in something called *Data as a Platform* (DaaP) - an integral component of population health management. Some of the examples of DaaP enhancing care quality are:-

- *Kanta* services is an interoperable national health data platform applied in Finland. This platform incorporates EHRs, e-prescriptions, investigations records (lab, imaging, biomarkers, etc), individual well-being, social care, and personal data. With all data protection and cyber security safeguards in place doctors and patients, can access the data anytime. Interoperability ensures that data can be shared seamlessly between the service providers but also overseas access in an event of an emergency when out of country [8].
- *Patients Know Best* (PKB) is an interoperable, secure personal health record (PHR) technology platform for patients in the United Kingdom (UK). This PHR pools together data from health and social care providers which is accessible to patients anytime enabling them to play a greater role in monitoring their health and its progress through special tools. Furthermore, PKB integrates with the National Health Services (NHS) app ensuring combined access of data to patients. The key highlight is the segregation of records such as - general, social care, mental, sexual, in terms of sensitivity to protect privacy. This also provides control in the hands of patients as to which data they intend to share with the provider [9].

### Connected care and population health: changing outcomes

The envisaged positive outcomes of connected care at the population level applies to the community setting as well as individual level.

- ***Community setting***

*Gesundes Kinzigtal* is a German town with a population of around 33,000 people. Since 2016, by exploiting an advanced data management system, and nurturing good relationships between network partners and communities, this model has demonstrated healthier and more cost-effective outcomes when compared to the overall population of the state. The IT infrastructure tracks patients across the system gather insurer, hospital, and provider data for analysis to stratify high-risk patients, predict and plan individualized intervention programs with outcomes [10].

*Dignio* is a cloud-based smart digital platform for medication that taps IoMT connected home monitoring devices collecting data like boold pressure, glucose, weight, temperature, sleep, physical activity, sleep, etc. and transmits them onto a patient’s smartphone through Bluetooth [11]. The medication dispenser called *Pilly SMS* contains 28 chambers for medication, sounds an alert, and releases medication at the schematized times. If the medication is not picked up from the device, patient notifications are delivered via SMS. *Medicio* (another dispenser) delivers the individualized kit of prescribed multiple medications. It sounds alert at the designated time and if the medication is not collected the health provider, family members, and the patients are informed via SMS. Both smart medication dispensers are integrated on Dignio platform. This connected care platform is tailored for usage in home as well as a hospital-based setting for both public and private sectors [11]. While successfully applied in Norway and Denmark, Dignio has resulted in positive outcomes for patients (39% lesser bed days in the hospital) and for clinicians (42% lesser doctor appointments).

- ***Personalized care***

Dubai-based *GluCare’s Integrated Diabetes Center* employs integrated continuous data monitoring for diabetes patients. A connected starter kit comprises a clinical-grade multisensory wearable band (which measures heart rate and its variability, respiration rate, physical activity, skin temperature, and sleep patterns), an app, and wearable continuous glucose monitors integrated on a clinical platform. The data is collected in real-time and monitored 24/7. Artificial intelligence (AI) and machine learning provide real-time insights and identify risk factors for co-morbidities for every patient so that medical interventions if needed, between clinic visits, could be done by the clinicians. The platform also obtains data inputs from other IoMT enabled technology for instance a smart weight scale, blood pressure cuff, exercise bike, etc. The app displays glucose readings, insulin dosages, tracks activity and sleep, logs meals, and ensures two-way communication between health coaches, clinicians, and patients [12]. This is a comprehensive and 24/7 real-time view of a patient’s connected care model which can easily be replicated in RMDs.

*My Arthritis DTx* app has been created to handhold patients with inflammatory arthropathies to self-manage their condition. Besides educational resources, the patients can utilize cognitive behavioural therapy, acceptance and commitment therapy, and mindfulness services. They can also connect with their clinical team and asynchronously communicate with them via the app. Users track their arthritis over time, monitor various parameters like physical health, mental health, sleep etc. [13].

### Big data driven transformation in RMDs care and research

Availability of myriad healthcare data, computing prowess, and algorithms are rapidly making the promise of AI a reality. Due to the advancements in deep learning algorithms can produce layers of abstract characteristics that allow computers to identify intricate concepts like diagnosis by constructing on simpler data pieces available in data such as radiographic images. The ability to learn and build upon distinctive attributes and features automatically imparts a higher level of accuracy with autonomy. Thus, explainable and causal AI is slated to play a good supportive role in clinical decision-making in the days to come. However, AI is a work in progress as far as making a groundswell and creating a significant impact is concerned. According to the recently published 2020 American College of Radiology Data Science Institute artificial intelligence survey, almost 34% of radiologist respondents indicated they use AI currently in their clinical practices, while 94% of the AI users reported that “the performance of AI in their practice was inconsistent” and barely 5.7% said that the technology “always works” [14].

*Sensely* is a US-based company that has made available a virtual nurse assistant for chronic disease management. These can be used for individualized monitoring and follow-up care. The virtual assistant can be accessed by the patients at home through digital devices in supporting their day-to-day care management and staying connected with their clinician. By using text-to-speech and speech-recognition technologies, a chronic-care platform for multiple diseases provides personalized conversational content to the respective patient. It helps guide the patient step-by-step through daily monitoring needs and assesses symptoms [15].

Cleveland Clinic and technology giant IBM have entered into a 10-Year partnership to accelerate research & discovery with the objective that the IBM hybrid cloud, high-performance computing, AI, and quantum computing technologies will provide foundation blocks for Cleveland Clinic Global Center in Pathogen Research & Human Health. This collaboration is a step towards the application of big data medical research and discovery for patient care. At the planned Discovery Accelerator advanced computational technology will generate and analyze data for enhancing research in areas such as genomics, single-cell transcriptomics, population health, clinical applications, and drug discovery [16].

*CreekyJoints* is a patient community initiative that comprises patient education resources including information such as how to manage chronic inflammatory conditions with Covid-19 imposed restrictions, accessing telemedicine, exercise, conversing better with doctors, etc. [17]. This is integrated with the *ArthritisPower* app which plugs into a patient-led research group. The app packs in individual disease tracking, analytics, medication tips, patient education, and other resources to manage the disease better. Also, another associated site – *eRheum* helps the patients in making and accessing telehealth at an individual level.

Furthermore, CreakyJoints and informatics researchers at the University of Alabama at Birmingham have come together to power a patient registry for adult RMD research [18]. Linked with EHR, laboratory results, and claims, the app collects active and passive data in structured and unstructured formats which include additional data feed on health tracking parameters (patient-reported outcomes [PROs]), medications, side effects, physical activities, QoL, etc. The registry infrastructure creates a vibrant ecosystem to conduct research. Based on the aforementioned data, patients are offered to participate in any newly initiated or ongoing study. This makes the recruitment process easy, cost-effective and time-saving besides the ease of constant monitoring during the study span. The participation in the general registry is available to the entire ArthritisPower community of more than 17,500 patients. So far, more than a dozen nested (ancillary) studies have been initiated with specific subgroups in sample sizes ranging from 110 to 1000 subjects [19].

### Data driven approaches and shortcomings

Real-world data (RWD) and its impact on various aspects of care delivery are well established [20]. The digital footprints (RWD) any patient or normal individual generates get compounded with the number of computing devices in use, the number of networks one keeps shifting into, and the accounts operated on devices in a single day. Unbeknownst to the patients, they are not aware of what disease-led data is being captured, where and with whom it is shared without their consent. Important issues which merit our attention are enumerated below:-.

#### Ethical and privacy concerns

Vast amount of data being generated brings forth ethical and privacy challenges. The concerns are compounded further as the data pieces are very personal in nature intrinsic or specific to the individuals. Besides the identity theft risks bigger threats are when accelerometer and global positioning system (GPS) enabled wearables to track gait, step counts, and exercise relay an individual’s location data over mostly unsecured networks. Invasion or theft of these data is not only a personal security risk but also a soft target for physical tracking by stalkers.

#### Data breach and cybersecurity

Data capture and transfer from medical devices, healthcare apps, implants, EHRs are increasingly being done over wireless connections. This makes them vulnerable to hacks and is potentially harmful to patients jeopardizing their safety. A woman in Germany seeking emergency care for a life-threatening condition died because the hospital came under ransomware attack and she had to be shifted out of the hospital [21]. About 28 million health records were compromised by hacking in the first half of the year 2019 alone [22]. The disruption to healthcare operations by hackers through remote access to the system was ranked as the top patient security risk in 2019 [23].

#### Data overload and digital overdiagnosis

A retrospective review by Wyatt et al. on patients evaluated for abnormal pulse over a period of 4 months with Apple watch yielded interesting results. Clinical documentation of 41 (15.5%) out of 264 subjects including patients, explicitly yielded an abnormal pulse alert. Pre-existing atrial fibrillation was noted in 58 (22.0%). Only in 30 (11.4%) patients who received an explicit alert did a clinically actionable cardiovascular diagnosis of interest was established. Thus false-positive results can cause unnecessary anxiety for the individuals as well as the waste of resources devoted towards investigations including clinicians’ valuable time [24].

### AI and clinical trials

**Fig. 3:**
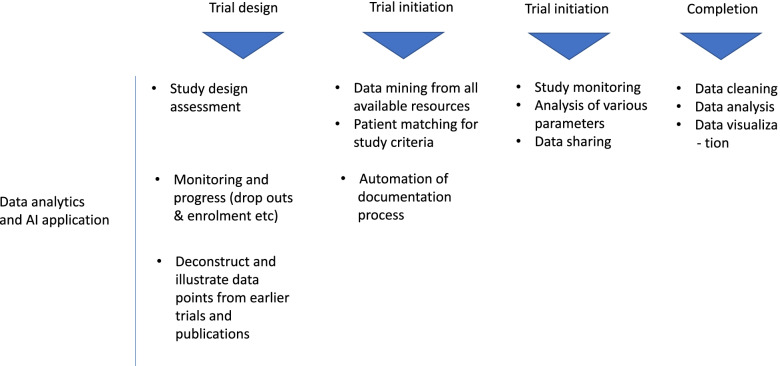
AI application in various stages of clinical trials. From authors own source, made using licensed version of MS Powerpoint

Data-driven algorithms with or without the combination of real-world data have the potential to benefit various aspects of clinical trials (Fig. 3) [25–27]. With the help of AI, patients are screened to check eligibility [28–30], predict the likelihood of patients to enroll [31, 32] or extract attributes EHRs [33–35]. For designing appropriate eligibility rules for clinical trials, an AI algorithm like Trial Pathfinder may come in very handy. Trial Pathfinder mines EHRs and scrutinizes the eligibility or exclusion of patients for the trial as per the laid out protocol. Upon application, the Trial Pathfinder had resulted in doubling the number of potential trial enrolees. It has also helped in broadening the patient pool to be more inclusive by bringing a greater number of women, minorities, and older patients [36]. From protocol design to study execution - AI has the potential to transform key steps in clinical trials [37].

### Flaws in datasets and their impact on AI

AI and machine learning are not yet part of the mainstream and remain at the fringes in clinical decision support for clinicians. Recently researchers at Mayo Clinic reported that the use of their AI-powered decision support tool based on electrocardiogram for predicting a low ejection fraction increased diagnosis of the condition by 32% in the routine primary care setting [38]. However, the long-term impacts on costs or outcomes have not yet been examined; therefore complete picture still remains elusive.

Partisan datasets lead to flawed inferences that potentially amplify race, gender, socio-economic variants. Researchers at Massachusetts Institute of Technology (MIT) identified that the 10 most cited AI data sets were riddled with labelling errors. To be able to map AI capabilities progression over time, researchers evaluate machine-learning models on core data sets. ImageNet is one of the best-known canonical image-recognition data set. The MIT research group found that ImageNet comprised of racist and sexist labels besides containing images of individuals whose consent was not obtained. Furthermore, many of the labels were grossly incorrect such as mushroom was labelled a spoon, a frog was labelled a cat, and the estimated label error rate was 5.8% [39]. In essence, wrong data labels lead to an inflated picture of brilliance for AI algorithms.

### Assistive and rehabilitation technologies in RMDs

**Fig. 4:**
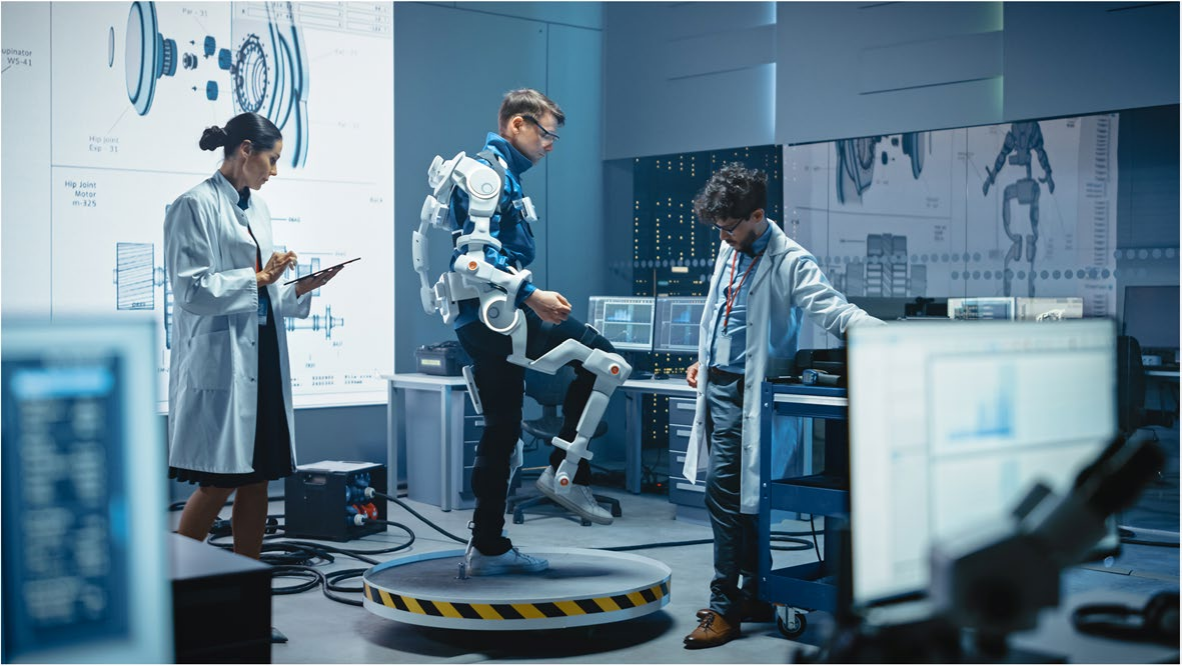
Exoskeleton and analytics. This Figure has been obtained from www.shutterstock.com under author’s corporate subscription plan. This allows author’s to use the images in any forum without any copyright restrictions

Researchers using the Cause of Death Ensemble model and Bayesian metaregression tool estimated 138.7 million disability-adjusted life years (DALYs) for 195 countries in 2017 due to musculoskeletal disorders [40]. Good health is required for individuals to have economic, social, and functional independence during their lifespan [41]. Technology is creating a significant impact in the lives of people living with chronic diseases such as RMDs and helping them lead more productive life [42]. For example, Exoskeleton is a wearable assistive device (Fig. 4) that alters the user’s limb-joint dynamics to reduce the user’s metabolic expenditure during natural level-ground walking and running as opposed to without an assistive device [43]. The aim is to reduce human endeavor. Metabolic cost is the gold standard to assess lower-limb exoskeleton performance. While relating closely to overall performance, the metabolic cost is an objective measure of effort within a given gait mode [44, 45].

Stanford University researchers have created an ankle exoskeleton system that is attached around the shin and into a running shoe. This enhanced the self-selected walking speed of individuals. Regulated by an algorithm, the exoskeleton was tested on 3 parameters for mobility, optimization for speed, optimization for energy use, and a placebo mode adjusted to make the subjects walk more slowly. Optimization for speed resulted in a 42% increase in walking pace and there was a reduction in energy use by 2% per meter travelled. The placebo mode slowed down the subjects and enhanced their energy use which was intended [46]. For patients impacted with mobility issues, this approach may reduce pain owing to the body’s weight on joints and/or also improve balance.

*ReStore* soft exo-suit is a garment-like design. In order to enable functional gait training activities, this adaptively synchronizes with a patient’s natural gait. By deploying real-time analytics the system allows therapists to adjust and optimize treatment. ReWalk Robotics has already obtained a CE mark and is a significant step in mobility and rehabilitation over rigid exoskeletons [47].

*GyroGear* has created a glove system with in-built gyroscopes to help patients suffering from “essential tremors” in regaining control of their hands. Parkinson’s disease and essential tremor result in severely impacting everyday tasks such as eating, drinking, dressing up because patients’ hands shake beyond control [48].

**Fig. 5:**
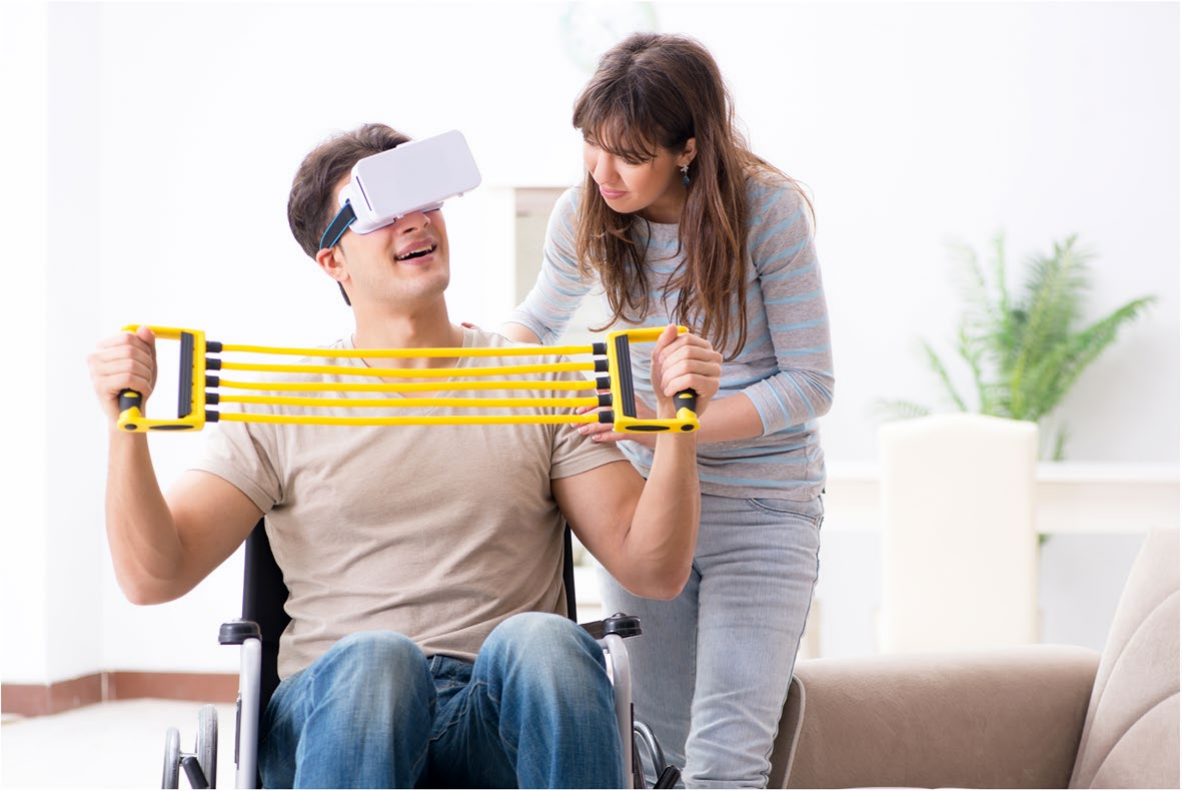
This Figure has been obtained from www.shutterstock.com under author’s corporate subscription plan. This allows author’s to use the images in any forum without any copyright restrictions

Virtual reality (VR) is being applied for different purposes, ranging from learning and development (cognitive outcomes), to mental therapies (emotional outcomes), to physical rehabilitation (physical outcomes). Utilizing the VR headsets a range of virtual physical therapy and rehabilitation options are being tried for patients (Fig. 5).

US Food and Drug Administration (FDA) has approved EaseVRx - VR system as a prescriptive chronic low pain treatment. This system comprises a VR headset and a device that amplifies the user’s breath sound to support breathing exercises. The principles from cognitive behaviour therapy are the basis of this system, which aims to help patients comprehend and interpret various thought patterns and emotions. Relaxation, distraction, and improved awareness of internal signals are deployed to address pain in this program. 179 individuals with low back pain (duration > 6 months) were enrolled and randomized 1:1 to 1 of 2 daily (56-day) VR programs: (1) EaseVRx (immersive pain relief skills VR program); or (2) Sham VR (2D nature content delivered in a VR headset). After enrolment, participants were followed for duration of 8.5 months in total. This included a two-week baseline assessment period, an eight-week VR program, a post-treatment assessment, and follow-up at one, two, three, and 6 months after completing the program [49].

66% of the EaseVRx group reported > 30% reduction in pain, compared to 41% of the control group who reported > 30% reduction in pain. 46% of the EaseVRx group reported > 50% reduction in pain compared to 26% of the control group. At one-month follow-up, the entire EaseVRx group continued to report a 30% pain reduction, and at the two- and three-month follow-up marks, the 30% reduction in pain was reported for all outcomes with the exception of pain intensity. While the control group reported a reduction in pain below 30% at one-, two-, and three-month follow-up for all outcomes [49].

High user satisfaction, superior and clinically meaningful symptom reduction for average pain intensity, and pain-related interference with activity, mood, and stress resulted with EaseVRx as compared to the control group [49].

We would like to highlight an important point herein that although rehabilitation through VR seems promising and is in the evolutionary stage the patient activities, resultant data, and its ownership are grey areas still. The right ownership of data whether it rightfully belongs to the patient, healthcare system or device manufacturer is still unresolved matters.

### The evolving role of 3-D printing

In healthcare, regenerative medicine and tissue engineering are being revolutionized by 3-dimensional (3D) bio-printing. Precision engineering for prosthetics and personalized pharmaceuticals are also being done through 3 D printing. By customizing the size and geometries of tablets and drug delivery devices through 3D printing technology they can be programmed to control their release and dosages based on clinical need.

#### 3D printing for medication

*Triastek* is a pharmaceutical and 3D printing technology company that has obtained Investigational New Drug (IND) approval from the US FDA for its 3D printed drug product, T19 which is designed to treat rheumatoid arthritis [50]. The tablets are constructed with sophisticated shapes and internal geometric structures by automated intelligent manufacturing. These unique structures allow drug delivery to be closely controlled and adjusted for onset time, duration, and mode for dissolution. This ensures more predictable and reproducible drug delivery results. Deploying 3D printing for T19’s novel design permits it to function as a chronotherapeutic drug delivery system. The premise of 3D printing investigational drug is by administering medicine at different durations of the circadian rhythm will maximize the drug’s therapeutic impact and minimize side effects.

Medication adherence is a global problem and it’s estimated that almost 50% of doses are missed [50, 51]. A group of researchers has created a 3D printer that builds several strata of medications in a single pill [51, 52]. The entire pill is printed out of finely powdered drugs and is stacked up with medicines layer by layer so that one drug could release quickly while the other releases slowly. The possibility of packing multiple medications in a single pill with different pharmacokinetics opens up interesting possibilities and may ensure greater patient compliance for medication.

#### 3D printing for prosthetics

Another research group has developed a ceramic ink that can be 3D-printed at room temperature with live cells devoid of chemicals [53]. The current gold standard for repairing bone is an autologous bone graft but a high rate of infection is an impediment to this process. The new technique could potentially be applied to print bone directly into a patient’s body. Also, if the required graft size is big then 3D printing is a perfect solution for customized manufacturing. Some of the interesting medical and research applications with 3D-printed bone tissue are, bone disease modelling, drug screening, bone microenvironment study, and damaged bone repair due to trauma, cancer, or other illnesses.

*REJOINT* creates customized knee joint prosthetics for patients by combining 3D printing using additive manufacturing and augments this with artificial intelligence [54]. The creation of tailored prostheses begins with 3D modelling of the patient’s computerised tomography (CT) scan. Algorithms analyze the images and determine the most qualified size for each unique case. The unique anatomy of a patient on several thousand prosthetic dimensions is compared through Artificial intelligence. For positioning the prosthetic components the optimal configuration is identified. The analytical process is the core of prosthesis production. The data-driven approach is the core of the process that eliminates minimal dimensional differences eliminating patient discomfort and other side effects like pain and inflammation commonly associated with knee arthroplasty.

## Conclusions

In this review, we have illustrated the potential benefits of data science, sensors, engineering, and computation to patient care and research in RMDs. We envisage that the following mechanisms when adapted for RMDs will enable optimum utilization of the data for the betterment of patient care,

- Creating processes for the use of big data collected through technology-enabled tools to facilitate a better understanding of rheumatological health, what it is, what causes/triggers immunological mechanisms, and what treatments are effective. Involving stakeholders whose data is being collected and used, regulating privacy and transparency in obtaining consent, is absolutely necessary.
- Using technology to enable wider and greater access to care, thus bridging the digital divide and promoting health equality.
- Adopting a practical approach to manage rheumatological health at the level of community through education, advocacy using technology for a multiplier effect, and addressing treatment gaps quickly.
- Using proactive interventions in the face of resource limitations and addressing health disparities by creating greater patient advocacy.
- Addressing data capture, data sharing, and interoperability to build data infrastructure in RMDs with multiple disease syndromes involving small or large patient groups.

## Data Availability

Not applicable.

## Abbreviations

WHO: World Health Organization
RMDs: Rheumatic musculoskeletal diseases
EHR: Electronic health records
IT: Information technology
IoMT: Internet of Medical things
QoL: Quality of life
DaaP: Data as a Platform
PKB: Patients Know Best
PHR: Personal health record
UK: United Kingdom
NHS: National Health Services
AI: Artificial Intelligence
PROs: Patient-reported outcomes
RWD: Real-world data
GPS: Global positioning system
MIT: Massachusetts Institute of Technology
DALYs: disability-adjusted life years
VR: Virtual reality
FDA: Food and Drug Administration
3D: 3-dimensional
IND: Investigational New Drug
CT: Computerised tomography
